# Effect of Germination on the Digestion of Legume Proteins

**DOI:** 10.3390/foods13172655

**Published:** 2024-08-23

**Authors:** Xinrui Wang, Bei Fan, Yang Li, Chengxin Fei, Yangyang Xiong, Lin Li, Yanfang Liu, Litao Tong, Yatao Huang, Fengzhong Wang

**Affiliations:** 1 Institute of Food Science and Technology, Chinese Academy of Agricultural Sciences, Key Laboratory of Agro-Products Processing, Ministry of Agriculture, Beijing 100193, China; wangxinrui202202@163.com (X.W.); fanbei517@163.com (B.F.); 13608175227@163.com (C.F.); 82101215459@caas.cn (Y.X.); lilin13259354082@163.com (L.L.); liuyanfang054@163.com (Y.L.); tonglitao@caas.cn (L.T.); 2 Western Agricultural Research Center, Chinese Academy of Agricultural Sciences, Changji 831100, China; 3 College of Food Science and Engineering, Qingdao Agricultural University, Qingdao 266000, China; liyangstrel@qau.edu.cn

**Keywords:** germination, legume protein, anti-nutritional factor, structure, digestion

## Abstract

As one of the main sources of plant protein, it is important to improve the protein digestibility of legumes. Faced with population growth and increasing environmental pressures, it is essential to find a green approach. Germination meets this requirement, and in the process of natural growth, some enzymes are activated to make dynamic changes in the protein itself; at the same time, other substances (especially anti-nutrient factors) can also be degraded by enzymes or their properties (water solubility, etc.), thereby reducing the binding with protein, and finally improving the protein digestibility of beans under the combined influence of these factors The whole process is low-carbon, environmentally friendly and safe. Therefore, this paper summarizes this process to provide a reference for the subsequent development of soybean functional food, especially the germination of soybean functional food.

## 1. Introduction

**Figure 1:**
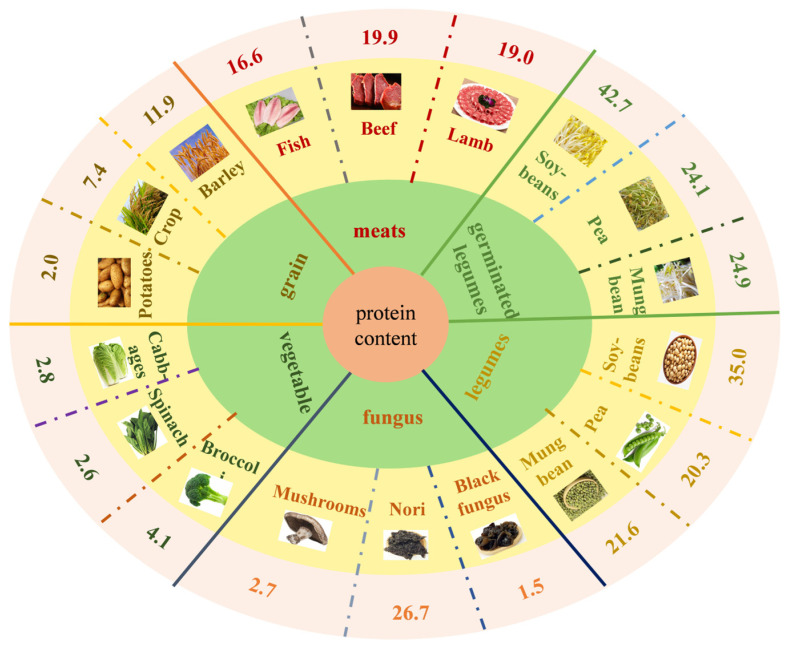
Comparison of protein contents of common animal protein, plant protein, and sprouted bean protein (g/100 g). All images used in the chart are sourced from the internet [43,44,45,46].

Proteins can contribute to substance metabolism by providing energy and essential amino acids, which are essential in humans [1,2]. Animal-derived foods are the primary protein sources for humans as they are rich in essential amino acids and are highly digestible. Due to population growth and environmental pressures, plant proteins have attracted increasing attention. As a result, food concepts are progressively moving toward plant-based foods [3]. As shown in Figure 1, according to the latest content of the Chinese food composition list and the literature description [4,5,6], compared with other vegetable proteins, the protein content of beans is higher and comparable to that of meat (beef, fish, lamb, etc.). However, the quality of a protein depends on its essential amino acid content and composition and also on its digestibility [7,8]. The digestibility of plant proteins is usually lower than that of animal proteins because of differences in digestibility and nutritional quality between animal and plant protein sources [9]. Soybean seeds contain about 40% dry-weight protein. They can be used as a high-quality, sustainable alternative protein source [10]; however, their reduced content of sulfur-containing amino acids (e.g., methionine) reduces their bioavailability to a large extent [9]. Therefore, improving the digestibility of legume proteins is essential to alleviate the shortage of plant proteins in the current environment.

The digestibility of proteins is closely related to the source and structure of the protein [4]. The looser the spatial structure of the protein, the easier its digestion. Hydrogen bonding is a major factor that hinders the enzymatic action of proteases on proteins; the lower the level of hydrogen bonding, the more favorable the protein digestion. Intermolecular and intramolecular disulfide bonds also determine the ability of trypsin to digest proteins; therefore, proteins with disrupted disulfide bonds are easier to digest [11]. Anti-nutritional factors, such as tannins [12], trypsin inhibitors [13], phytic acid [14], and lectins [15], are major factors in the reduction in protein digestibility as they bind to proteins, digestive enzymes, etc. Consequently, many studies have used physical [16,17], chemical, and biological methods [18] to investigate the effects of protein structural changes and anti-nutritional factors on protein digestibility. However, in the face of increasing population and environmental pressure, a green approach is particularly important. And sprouting meets that requirement. Germination means the end of dormancy, the maintenance and release of which is primarily determined by the synthesis and catabolism of endogenous abscisic acid (ABA) in the seed, in addition to other phytochemicals such as auxin, ethylene cytokinin, and gibberellin (GA) [19]. In the absence of other factors, the release of seed dormancy depends on the ABA/GA ratio, and the regulation of ABA and GA in seed dormancy and germination is antagonistic and dependent on the degradation of DELLA-proteins, which ultimately leads to the synthesis and secretion of hydrolase (Enzyme Commission class 3 enzymes, which include the amylases and proteases). These hydrolases promote the catabolism of endogenous nutrients in seeds, serve as an energy source, and provide components needed for synthesizing other compounds. Germination can increase the growth of peanuts [20], sesame [21], quinoa [22], barley [23,24], beans [11,25,26,27], and other cereals in terms of protein content, change the shape and composition of subunits, and improve protein properties. Among them, the protein content of common beans (soybeans, mung beans, and peas) can be increased by about 10% after germination (Figure 1); this may be related to changes in the enzymes mentioned earlier. At the same time, this can reduce anti-nutritional factors in soybeans (soybean, black bean [28], green bean, etc.) and mixed beans (chickpea [3,29,30,31,32], fava beans [5], kidney beans [33], cowpeas [34], lentils [29], peas [5,35,36,37], mung bean, etc.)), with relatively more studies performed using soybean (mainly soybean) and chickpea. As shown in the studies above, during growth and development, stored proteins are reactivated in the form of amino acids and energy needed for protein synthesis and growth, leading to changes in their content and structure [4,38]. In addition, other components of legumes, especially anti-nutrients, are degraded by enzymes (such as phytase) and by their own properties (such as water solubility). At present, there are patents for the preparation of sprouted soybean milk [39], sprouted chickpea konjac powder [40], and functional (rich in folic acid [41], γ-aminobutyric acid [42]) soybean sprout products. The results showed that the quality of the products made from sprouted beans was better than that from unsprouted beans.

Overall, germination is a green and effective approach to improve protein digestibility. This role is multifaceted and integrated; however, comprehensive discussions and analyses of its influencing factors are scarce. Therefore, this study sought to comprehensively analyze the effect of germination on the changes in legume protein and anti-nutritional factors and evaluate the mechanism of its influence on the digestive process to provide more theoretical support for the improvement of legume protein digestibility.

## 2. Changes in Protein during Germination

### 2.1. Changes in Protein Content during Germination

**Figure 2:**
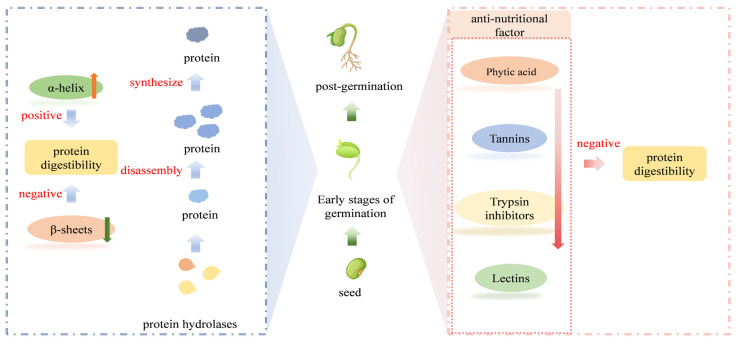
Changes in protein and anti-nutritional factors during soybean germination in relation to protein digestibility. Bean pictures were sourced from the Internet [52]; the rest are original.

At the early stage of germination, endogenous proteases are synthesized and activated, and stored proteins are continuously cleaved and broken down by protein hydrolases (e.g., peptidases such as cysteine endopeptidase, serine endopeptidase, serine carboxypeptidase, aspartate endopeptidase, aminopeptidase, and cytoplasmic dipeptidase; and proteases such as aspartate protease, protease C1, protease C2, protease B, and protease F), which provide energy for the growth and development of the organism [47,48]. As germination proceeds, novel proteins will be synthesized; therefore, protein changes dynamically during the germination process (Figure 2).

Increased protein levels were reported to increase to approximately 4% after the germination of soybeans at 25 °C for seven days in the dark [49]. The increase in protein content during germination may be due to a number of factors, such as the synthesis of enzymes during protein synthesis leading to the production of certain amino acids, as well as the catabolism of various components and the production of simple peptides during the degradation of protein macromolecules. It was mentioned that the synthesis of storage proteins slowed down with increasing germination time, while the genes encoding protein degradation (peptidases and proteases) were up-regulated [47]. After 36 h of germination, the protein content of ZD41, J58, and JHD soybean varieties decreased by 0.56%, 4.02%, and 0.70% with the extension of germination time, and the water-soluble protein content increased by 30.52%, 9.34%, and 10.97%, respectively. This may be because soybean protein may break down into smaller molecules, thus increasing the degree of hydrolysis [47]. Although no significant increase in crude protein content was observed in this study, the increase in protein solubility should favor the nutritional improvement of short-term germinated soybean seeds. The solubility of proteins is not only related to hydrophobicity but also to ionic interactions and temperature. The more anions there are, the more favorable it is to increase the solubility of proteins; at the same time, in the range of 0–40 °C, the higher the temperature, the higher the solubility. However, other studies have observed no significant change in protein content during germination of soybeans [50]. Legumes may show different changes in protein content during germination owing to differences among species and the balance between protein degradation and biosynthesis [51].

### 2.2. Changes in Protein Structure during Germination

A previous study showed that the proportion of anti-parallel β-sheets and β-sheets increased with the progress of germination, while the proportion of α-helices and β-turns decreased. This suggests that the protein structure of small lentils becomes looser during germination (Figure 2) [25]. The decrease in α-helices and the increase in β-sheets in lentils during germination may lead to an increase in surface hydrophobicity. In addition, studies have shown that surface hydrophobicity was positively correlated with β-sheets and negatively correlated with α-helices [53]. The intestinal transporter carrier PepT1 is the main mode of proteolytic peptide transport in the gastrointestinal tract, and the hydrophobicity of the peptide determines its affinity for PepT1 (Table 1). The sites involved in the binding of dipeptide and tripeptide in small peptide transporters are conserved hydrophobic amino acids, and residues on these transporters tend to bind more hydrophobic side chain peptides [54,55,56,57]. The β-turns usually occur near the surface of the protein, where the peptide group of the middle two amino acid residues can form a hydrogen bond with water. Thus, a reduction in β-turns indicates that germination results in changes in surface structure and interaction [26].

In addition, Sofi, et al. [11] and Sun, et al. [26] used SDS-PAGE to study the changes in the composition of soybean protein subunits during germination. The results showed that the bands of α and α′ subunits of β-conglycinin (7S) and acid subunits of Glycinin (11S) gradually disappeared with germination time, but the bands of β subunits of 7S and alkaline subunits of 11S did not change significantly. The peaks at 47, 40, 25, and 24 kDa were attributed to the subunits of legumin, the peaks at 50, 35, 33, 19, 15, and 13 kDa were attributed to the subunits of vicilin, while the peak at 55 kDa was attributed to glutelin and 12 and 10 kDa were attributed to the subunits of albumin [64,65]. The 7S and 11S subunits are connected by disulfide bonds, and a reduction in their content indicates that disulfide bonds are broken and reduced to free sulfhydryl groups, making the protein structure looser [21]. All these above data indicate that germination can change protein structure, but the changes in protein tertiary structure during germination and the specific mechanism of these changes are rarely reported.

## 3. Changes in Anti-Nutritional Factors during Germination

### 3.1. Changes in Tannins during Germination

Tannins are unique high molecular weight phenolic metabolites that form strong complexes with carbohydrates and proteins and can be classified as soluble or insoluble based on their structural uniqueness. Soluble tannins include oligomeric proanthocyanidins and hydrolyzable tannins of relatively low molecular weight, which are readily soluble in water or organic solvents (e.g., methanol, acetone, etc.) [66]. Red kidney beans soaked in reverse osmosis water at 25 °C for 24 h reduced tannin by 74% [67]. Cowpeas soaked in water, and 0.03% sodium bicarbonate solution for 6 h reduced their tannins by 7% and 8%, respectively [18]. A similar study found that soaking cowpeas in 0.05 g/100 mL sodium bicarbonate solution for 12 h and then boiling for 30 min reduced tannin content by 76% [34]. This indicates that soaking the seeds just before germination is effective in reducing the tannin content. After 24 h of germination, the total polyphenol and tannin contents of mung beans were reduced by 43% and 59%, respectively [68]. During the germination period (24, 48, and 72 h), the tannin contents of chickpeas decreased by 14.4%, 18.5%, and 43.4%, respectively [69]. After germination at 60 °C for 72 h, the tannin content of broad beans decreased by 20% to 53% [70]. According to the researchers, this result may be due to an increase in the activity of endogenous enzymes during germination.

### 3.2. Changes in Trypsin Inhibitor during Germination

Legumes contain approximately 2% trypsin inhibitors, mainly Kunitz trypsin inhibitors (KTI) (1.4%) and Bouman–Birk trypsin inhibitors (BBI) (0.6%). Of these, KTI is considered harmful to human health, whereas BBI has positive anti-tumor effects [71]. Based on the structure of KTI (which comprises two disulfide bonds and a reaction site for trypsin (EC 3.4.21.4) or chymotrypsin (EC 3.4.21.1) and is heat labile) [72], many scholars have found that physical (heat treatment [73], high-pressure treatment [74]), chemical [75], and biological (fermentation, germination) methods [76] can be used to reduce trypsin inhibitor levels. Germination is an environmentally friendly and effective method. Studies have shown that trypsin inhibitor levels in cowpeas decreased by 11%, 16%, 17%, and 19% during germination (24, 48, 72, and 96 h, respectively) [34]. The trypsin inhibitor levels in soybeans decreased by 22.3% after germination for 3 d at 25 °C [74]. Another study found that when the soybeans germinated in 27 °C darkness for 4 days, trypsin inhibitor levels decreased by 22.3% [77]. In addition, trypsin inhibitor activity was reduced by 76% in white kidney beans germinated under light conditions for 7 d [76]. This phenomenon could be due to the presence of hydrophobic reductases and specific hydrophobic proteases in germinating legume seeds, which enable the disruption of disulfide bonds in the trypsin inhibitor molecules under environmental conditions, leading to the inactivation of the trypsin inhibitor via the disruption of the active centers of the trypsin inhibitor, and the seed enzyme system that can disrupt and break down proteinaceous anti-nutritional factors during the germination process [78].

### 3.3. Changes in Phytic Acid during Germination

According to previous studies, phytase is the most common source of phytate hydrolysis enzymes, which can effectively reduce phytic acid content and improve the digestion and absorption of proteins and other nutrients [79,80,81]. Monogastric mammals (e.g., humans and pigs) lack phytase in the digestive tract; therefore, increasing phytase activity in food is an important part of the research; however, grain phytase is safer and more acceptable to consumers than added bio-enzymes [82]. Germination treatment has been shown to increase phytase activity by 3–5 fold [83], indicating its effectiveness at reducing phytic acid content. After germination at 30 °C for 120 h, the phytic acid content of the broad beans decreased from 9% to 69% [84]. The phytic acid content of three kinds of kidney beans (*Roba*, *Awash*, and *Beshbesh*) decreased from 23.51 mg/g, 24.06 mg/g, and 17.34 mg/g to 1.88 mg/g, 5.06 mg/g, and 0.69 mg/g, respectively, after four days of germination, with an average decrease of more than 75% [85]. In addition, some researchers germinated lentils for six days under alternate watering in a dark room and found an 82% reduction in bound phytic acid [82]. Ultimately, in the presence of phytase, phytic acid is broken down to produce free inorganic phosphorus and intermediate inositol phosphate chains (inositol pentaphosphate to inositol monophosphate) in its Myo-inositol ring, which provides more phosphorus for bone growth [79].

### 3.4. Changes in Lectins during Germination

Lectins are tetramers (consisting of four subunits) and high-affinity glycoproteins, with a content of approximately 3% in legumes. Most lectins have an anti-digestive effect on proteases in the body and even have the undesirable effect of irritating the intestinal wall and hindering the digestion and absorption of nutrients. Therefore, lectins are considered to be a type of anti-nutrient, and eliminating their anti-nutritional properties is a concern in the field of food processing. Studies have shown that lectins are primarily present in the seed coat and can be completely removed by soaking and steaming treatments [86], with germination as a good method [49,87]. The lectin content of dark red beans decreased from 7% to 18% after 4 d of germination at 25 °C, and that of white kidney beans decreased by 85% after 7 d of germination at 20 °C [76]. However, only a few studies have recently been conducted on the relevant changes in lectins during germination, and the specific mechanisms underlying these changes are unclear.

## 4. Effect of Changes in Proteins and Anti-Nutritional Factors on Legume Protein Digestion

Germination improves protein digestibility in millet [88], sorghum [89], chickpea [3], and cowpea [90], among others, because during the process of germination, a large number of hydrolytic enzymes are released or synthesized, which degrade anti-nutritional factors or hydrolyze biopolymers (e.g., proteins) [91,92].

### 4.1. Effect of Structure on Protein Digestibility

High molecular weight proteins, lower molecular weight proteins, and other smaller molecules that are more readily absorbed by the body during germination suggest that soybean sprouts are better than soybeans from a nutritional point of view [26]. Similar phenomena were reported by Zinia, et al. [93], Ohanenye, et al. [4], and Wang, et al. [58] suggested that germination could enhance endogenous enzyme activity to degrade some large-molecule proteins into small-molecule substances and improve protein digestibility. According to previous studies, the α-helix content was observed to be positively correlated with protein digestibility in vitro, while the β-sheet content was negatively correlated [17,94,95]. Although relatively few studies have been conducted on the structure-digestion properties of germinated soybean proteins, numerous previous studies have shown that changes in the spatial structure and subunit composition of soybean protein under treatments, such as “heat treatment [17,96], ultrasonic treatment [97,98], deamidation and ultrahigh-pressure treatment [99]” have a significant effect on the protein digestibility in vitro, as heat treatment and ultrasonic treatment can reduce the protein digestibility in vitro, by cross-linking disulfide bonds, while deamidation treatment can reduce the content of disulfide bonds and improve the protein digestibility in vitro. A positive correlation between α-helix content and protein digestibility in vitro and a negative correlation between β-sheet content and protein digestibility in vitro were found under heat treatment, ultrasonic treatment, and deamidation treatment. In vitro protein digestibility decreased as hydrophobic and electrostatic interactions increased and increased as hydrogen bonding decreased.

In the natural state, the unique amino acid composition of proteins causes them to be folded into a certain conformation, and this tightly folded conformation can hinder protein digestion [61]. In addition, protein bodies are encased in cellular structures to form supramolecular structures that further restrict the hydrolase’s access to the substrate [62,100]. Methionine (Met) deficiency in legumes has resulted in less than 60% true utilization of legume proteins by humans. With germination, the ratio of essential amino acids to non-essential amino acids changes, which can provide further essential amino acids [6]. Research shows that germination can lead to a small increase in the essential amino acid methionine (Met), while the conditional essential amino acid cysteine (Cys) [26]. However, the quality of proteins should be considered not only in terms of their amino acid composition but also in terms of the digestion and absorption of their hydrolysates in the human gastrointestinal tract. The nutritional value of proteins from different sources varies considerably, and proteins with a good amino acid composition may also be poorly digested and/or absorbed [8]. A widely used method for evaluating the nutritional value of food proteins is the amino acid score (AAS), but it does not take into account the digestibility of food proteins. In recent years, the Food and Drug Administration (FDA) of the United States of America (US) has proposed a new method, i.e., the amino acid score corrected for digestibility (PDCAAS) (Table 1) [61,101]. The PDCAAS values for soybeans were about the same at 0.91 and beef (0.92), respectively [102]. Recently there has been a protein quality score considered superior to PDCAAS, the Digestible Essential Amino Acid Score (DIAAS), which is a method whereby a reference protein has an indispensable amino acid sequence similar to that required by children aged 0.5 to 3 years, and the amount of all digestible essential amino acids in the protein is compared with the amount of these digestible amino acids in the reference A method of comparing the amount of these digestible amino acids in a protein that utilizes the true ileal digestibility of the protein rather than fecal digestibility [63,103]. In addition to amino acids, as shown in Table 1, peptides are also an important form of amino acids absorbed by the body from proteins. The main pathway for the body to absorb peptides is PepT1, and its transport capacity is related to the molecular weight of the peptides. Peptides with molecular weight less than 0.5 kDa, including dipeptides and tripeptides, are transported to small intestinal epithelial cells for uptake via PepT1. The smaller the molecular weight, the easier it is to be absorbed, so the greater the proportion of small peptides in the enzymatic product, the easier it is to be digested and absorbed by the body [55,56]. Amino acids, peptides, and proteins that are not absorbed in the small intestine end up in the colon and are then metabolized by the microbiota [61,63].

### 4.2. Effect of Tannin on Protein Digestibility

**Figure 3:**
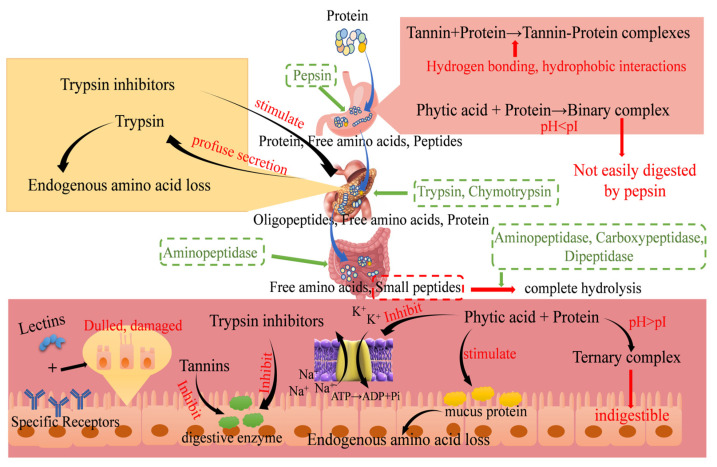
Protein digestion and inhibition of protein digestion by anti-nutritional factors. Gastrointestinal pictures were sourced from the Internet [112,113,114]; the rest are original.

For thousands of years, it was widely believed that tannins in the human diet were harmful or toxic to animals. Some studies have found that tannins inhibit protein digestibility by up to 28% [4]. The main mechanism is binding to proteins through hydrophobic interaction into the hydrophobic pocket of proteins; thereafter, the phenolic hydroxyl group of tannins binds to the polar groups of proteins (peptidyl, carbonyl, guanidinium, hydroxyl, etc.) through hydrogen bonding to form a tannin–protein complex, which hinders the digestion of proteins (Figure 3) [104]. However, the diversity of tannin structures and their dose-dependent and species-specific effects have been ignored by many researchers [105]. There is also research that shows grass carp could tolerate 1.75% dietary tannin without influencing growth; however, 1.25% tannin impaired the digestion and metabolism of proteins, decreased lipid deposition, and promoted the utilization of carbohydrates, hepatic glutamate aminotransferase and aspartate aminotransferase activities were also found to decrease with increasing dietary tannin levels, indicating impaired protein biosynthesis [105]. Glutaminase and aspartate aminotransferase are the major hepatopancreatic aminotransferases that break down amino acids absorbed in the intestine and convert them to α-keto acids [106]. Hydrolyzable tannins are readily hydrolyzed in the intestine to small phenolic compounds that are absorbed and may cause hepatopancreatic toxicity [107]. A single subcutaneous injection of 700 mg/kg bw of tannin to mice resulted in a significant breakdown of polysaccharides in the liver and inhibited the binding of amino acids to hepatic proteins [108]. So, the anti-nutritional function and toxicity of tannins may be related to this. In addition, tannins can form hydrophobic layers between protein molecules through multi-point binding, causing protein molecules to aggregate and precipitate [58]. It also inhibits the activity of amylase and trypsin, thus inhibiting protein digestion [109]. The inhibitory effects of low tannin and high tannin sorghum grains on α-amylase, trypsin, and lipase levels of rabbits were 37%, 77%, 22%, 56%, 6%, and 43%, respectively [110]. The activities of trypsin, chymosin, and α-amylase in jejunum could be inhibited by feeding salt seed meal containing tannic acid to broilers [111].

### 4.3. Effect of Trypsin Inhibitor on Protein Digestibility

Trypsin inhibitors are the main anti-nutritional factors in legumes that can impede the action of protein hydrolases in the intestinal tract and reduce protein digestibility. Approximately 14% of soybeans containing more than 4 mg/g of trypsin inhibitor used in the food industry can reduce the digestibility of proteins and amino acids [115]. The research found that trypsin inhibitor activity had the most significant effect on lupin protein digestibility, and low trypsin inhibitor activity was more favorable for protein digestion [13]. Meanwhile, the presence of trypsin inhibitors was negatively correlated with the ability of animals to digest proteins as the trypsin inhibitors in soybean bound to and inhibited the protease activity of pancreatically secreted trypsin and rennet [115]. Additionally, trypsin inhibitors can act on the pancreas itself, and trypsin in the intestine binds to the inhibitory factor and reduces the concentration of trypsin through excretion in the feces, stimulating the pancreas to secrete pancreatic enzymes in large quantities to achieve a compensatory response. This causes hyperfunction and a lack of endogenous essential amino acids secreted by the pancreas, especially methionine (Met), thereby causing dysfunction or disorder of digestion and absorption and reducing the digestion of protein in humans or animals (Figure 3). The apparent ileal digestibility of amino acids and crude protein was reduced by 13.3% to 26.0% and 23.3% when 38% soybean meal containing 8.78 mg/g of trypsin inhibitor was added to pig feed compared with soybean meal with 2.51 mg/g of trypsin inhibitor [116]. The research has found a quadratic effect of trypsin inhibitor levels on chick body weight and feed coefficient and a positive correlation with the relative weight of the pancreas [117].

### 4.4. Effect of Phytic Acid on Protein Digestibility

Owing to its distinctive chemical structure, phytic acid facilitates the formation of complexes with proteins and other substances, thereby lowering enzyme activity and protein digestibility [118,119]. The phytic acid may form complexes with proteins and protein hydrolases (pepsin and trypsin), resulting in the absorption and utilization of phosphorus, calcium, protein, and other phytate-bound nutrients in poultry that lack endogenous phytase [81]. Phytic acid can form complexes with proteins under both acidic and basic conditions. Phytic acid acts as a proton donor and produces hydrogen ions and “phytate” anions [82]. The “phytate” anions can bind to positively charged protein functional groups through multivalent cation bridging or indirectly to negatively charged protein functional groups [67]. At pH values below the isoelectric point of proteins, binary protein–phytate complexes are formed, which are not easily digested by pepsin. When the pH value exceeds the protein isoelectric point, the binary complexes dissociate, and ternary protein–phytate complexes begin to form and bind to proteins and peptides in the small intestinal chyme, affecting protein structure, which can reduce enzyme activity, protein solubility, and protein hydrolysis digestibility, hindering protein digestion and amino acid absorption in the small intestine [120]. In addition, phytic acid can stimulate mucus protein secretion, cause endogenous amino acid loss in the intestine, and promote the transition of Na^+^ to the lumen of the small intestine, leading to a decrease in the activity of the Na^+^-dependent transport system and sodium pump (Na^+^-K^+^-ATPase) and interfering with the absorption of glucose and amino acids in the small intestine [121]. Phytates contain 6 $HPO_{4}^{2-}$ molecules, which are strongly oxygenophilic and can indirectly interact with proteins by reacting with the surrounding aqueous medium, affecting protein digestibility (Figure 3) [122].

### 4.5. Effect of Lectins on Protein Digestibility

Lectins can enter the circulation of the body through intestinal epithelial cells, causing growth inhibition, organ damage, and even death, and are considered to be the main anti-nutritional factor in legumes [123]. According to its structural characteristics, lectins can form specific binding with N-acetyl-D-galactosamine or galactose [124]. This binding does not target sugar molecules in plant cells but the surface of microbial or animal cells [15]. Therefore, lectins not removed from food will bind to the surface of intestinal epithelial cells, affect the proliferation and differentiation of epithelial cells, destroy the brush marginal membrane, induce the shortening or dulling of villi, interfere with the digestion and absorption capacity of intestinal cells, and negatively affect the secretion and absorption of mucus in the digestive system (Figure 3) [125,126]. The research found that soybean lectins can inhibit growth and induce changes in the morphology and function of the small intestine and pancreas that are detrimental to intestinal digestion and absorption [127]. They investigated the effect of moderate doses of purified soybean lectins on nitrogen digestibility in rats and showed that fecal and urinary nitrogen losses increased from 0.13 + 0.03 g and 0.14 + 0.04 g to 0.24 + 0.03 g and 0.26 + 0.08 g when feeding diets without and with (0.4 mg/g) lectins, respectively. Loss of nitrogen in feces and urine reduces apparent protein digestibility and protein utilization. Soybean lectins can use their glycoprotein properties to bind to the carbohydrate chains of glycoproteins and glycolipids in the small intestinal membrane, reducing the chances of protein hydrolysis by the enzymes acting on soybean lectins and protecting it during passage through the small intestine. In addition, its binding to goblet cells on the intestinal mucosa induces a high secretion of mucus and binds to the intestinal epithelium, leading to a reduction in intestinal and brush border enzyme activity, all of which reduces nitrogen balance and retention [128]. Many other studies have also confirmed the negative effects of lectins on digestion, such as the addition of 0.1% to 1.0% rapeseed lectin (phytohemagglutinin) and 0.75% to 0.027% soybean lectins to the diet caused villi atrophy, increased crypt depth, and increased intestinal weight in the intestinal epithelium of rats [129]. Rats injected with lectins (50 mg/kg) for 6 weeks (3 times per week) were found to have small intestine atrophy, villi atrophy, and crypt hyperplasia, increased intestinal mucus and permeability, and decreased apparent ileal digestibility of crude protein [130]. At the same time, some studies show higher levels of morphological changes in the intestinal microvilli in animals fed soybeans containing lectin + trypsin inhibitor (KTI type) than in animals fed soybeans without lectin + trypsin inhibitor (KTI type), indicating that the levels of lectin and trypsin inhibitors were negatively correlated with soy protein digestibility [128]. Changes in the anti-nutritional factors during germination and their effects on protein digestion are shown in Table 2; after germination treatment, the anti-nutrition factors were reduced by about 70–80% on average. This is related to changes in enzymes during germination and their own properties (such as water solubility). Although the above studies did not explicitly mention whether the change in these anti-nutrient factors and the change in protein content and structure had a single effect or a synergistic effect on protein digestibility, according to literature data, we believe that it is a synergistic effect, but this needs to be proved by subsequent studies.

## 5. Summary

In summary, the effects of germination on the digestibility of legume proteins were compiled. In a study on the effect of the protein itself on digestibility, the protein content displayed a dynamic trend during germination. The variety, origin, size, storage period, and storage method of the germination, as well as the germination process (temperature, light, humidity, germination time, and whether the seeds were pre-soaked), all made a difference in the results of the studies (Table 3). Different bean varieties, temperature, pre-treatment methods (such as soaking), humidity, and germination time will cause different degrees of changes in protein solubility, hydrophobic interaction, ion interaction, etc., which will combine to lead to differences in protein digestibility during germination.

However, no correlation was found between the changes in protein content and protein digestibility. Of note is that the protein structure can affect in vitro digestibility through the secondary structure, hydrogen bonding, disulfide bonding, hydrophobic interactions, and electrostatic interactions. In the early stages of germination, the hydrogen and disulfide bonds in the protein structure are broken. The proportion of anti-parallel β-sheets and β-sheets increases while that of α-helices and β-turns decreases. In addition, the protein structure becomes looser and more disordered, which is more favorable for digestion. The content of α subunits, α′ subunits, and acidic subunits in 7S and 11S connected by disulfide bonds are reduced; however, the correlation between α subunits, α′ subunits, and acidic subunits, and protein digestibility remains unclear.

Anti-nutritional factors, such as tannins, phytic acid, and trypsin inhibitors, can bind to proteins and digestive enzymes and form complexes that are difficult to break down by digestive enzymes. Trypsin inhibitors and lectins can inhibit protein digestion by stimulating the pancreas or impairing the gut by binding to specific receptors on intestinal epithelial cells; however, their effects on protein digestion are also influenced by changes in the structure, properties, and associated enzyme systems. Oligomeric proanthocyanidins and hydrolyzable tannins of relatively low molecular weight, as well as lectins, are highly water-soluble and are broken down in large quantities during the early stages of germination. The KTI-type trypsin inhibitor that hinders protein digestion has a structure with two disulfide bonds and a reaction site for trypsin (EC 3.4.21.4) or chymotrypsin (EC 3.4.21.1). During the early stages of germination, hydrophobic reductases, and specific hydrophobic proteases disrupt the disulfide bonds in the trypsin inhibitor molecule, resulting in a decrease in the content. Phytic acid gradually breaks down as the phytase activity increases during germination. The reduction in these anti-nutritional factors (tannins, trypsin inhibitors, phytic acid, and lectins) significantly reduces their binding to proteins and digestive enzymes and reduces the stimulation of the corresponding digestive organs, thereby improving protein digestibility.

Germination significantly improves protein digestibility. As a result, germination is important to improve the utilization of legume proteins, reduce food consumption, and, more importantly, improve human nutritional health. However, studies on the effects of multiple component interactions on legume proteins during germination are lacking, and the processing and utilization of germinated legume proteins are worthy of further studies. As for the relationship between protein structure and in vitro digestibility, not only the digestibility itself but also an in-depth study of peptidomics after enzymatic digestion is important to enable a more effective explanation of the relationship between protein structural changes and in vitro digestibility. This information is crucial for the research and development of legume germination and related functional products.

## 6. Conclusions

With the growth of the population and an increase in environmental pressure, plant protein has attracted more and more attention. As the main source of plant protein, legumes are of great importance in improving the digestibility of legume protein in a green and environmentally friendly way to alleviate the shortage of plant protein in the current environment. The combination of factors such as the change in the protein itself and the reduction in anti-nutrient factor content during germination can effectively improve the digestibility of protein, and the germination treatment is more environmentally friendly than other physical, chemical, and biological treatments. Therefore, it is vital to study the influence of multi-component interaction on the germination process of legume protein and the mechanism of improving protein digestibility, as well as the research and development of functional soybean germination products.

## Figures and Tables

**Table 1:** Common evaluation indicators of protein digestibility.

Indicators	Methods	Reasons	References
particle size distribution	(1) Zeta potentiometer. (2) Laser particle size analyzer	The larger the average particle size of the enzymolysis, the higher the degree of molecular aggregation, the larger the molecular weight of the unenzymolysis, and the more peptides involved in the polymerization	[10,56]
degree of hydrolysis	(1) Fluorescent amine method. (2) OPA method	The release of free amino acids was measured	[58]
Surface hydrophobic index	ANS fluorescent probe method	The intestinal transporter carrier PepT1 is the main mode of proteolytic peptide transport in the gastrointestinal tract, and the hydrophobicity of the peptide determines its affinity for PepT1.	[55,56,59]
SDS-PAGE	SDS-PAGE	The protein in the digestive fluid was isolated and quantified.	[10,60]
molecular mass	(1) HPLC. (2) 18-angle laser particle size analyzer	Peptides with large molecular weight are difficult to absorb, so the more small peptides in the enzymatic hydrolysis products, the easier it is to be digested and absorbed by the human body.	[55,57]
Amino acid score (AAS)	AAS = Nitrogen or protein amino acid content (mg) per gram of food protein under test/Nitrogen or protein amino acid content (mg) per gram of reference protein × 100	A widely used method for evaluating the nutritional value of food proteins.	[8,61,62,63]
Protein digestibility corrected amino acid score (PDCAAS)	PCDAAS = Amino acid score × true digestibility	The US Food and Drug Administration (FDA) has come up with a new approach.	[8,61,62,63]
Digestible Essential Amino Acid Score (DIAAS)	DIAAS = mg digestible amino acids per gram of dietary protein/mg digestible amino acids per gram of reference protein × 100	The content of all digestible essential amino acids in the protein is compared with the content of these digestible amino acids in the reference protein.	[8,61,62,63]
Protein digestibility	The nitrogen value was determined by the AOAC(2005) method, and IVPD was the percentage of protein in the supernatant/the total protein content of the sample	Digestibility reflects the degree of protein degradation at different stages of the process.	[7,21]

**Table 2:** Changes in the anti-nutritional factors during germination and their effects on protein digestion.

Name	Reduction (%)	References	Effect on Protein Digestibility	References
Tannins	Reduced by 20% to 75%	[18,67,68,69,70,131,132]	(1) Binding with protein to form a complex. (2) Binding with digestive enzymes and inhibiting digestive enzyme activity.	[58,109]
trypsin inhibitors	Reduced by 10% to 80%	[34,74,76,77,115]	(1) Blocking protease hydrolysis. (2) Acting on the pancreas itself, it stimulates the pancreas to secrete a lot of pancreatic enzymes in a compensatory response, causing hyperfunction.	[13,116,117]
phytic acid	Reduced by 36% to 82%	[67,82,85,121]	(1) Binding with proteins or enzymes to form phytate-protein complexes and phytate-enzyme complexes. (2) Inhibit sodium pump activity and interfere with intestinal digestion of proteins as well as amino acid absorption. (3) Phytate interacts with protein.	[118,121,122]
lectins	Reduced by 7% to 85%	[49,76,87]	Specific recognition with N-acetylgalactosamine and galactose in intestinal epithelial cells damages the intestine, interferes with the secretion of intestinal digestive enzymes, and inhibits protein digestion.	[126]

**Table 3:** The increase in protein digestibility of different varieties of legumes under different germination conditions.

References	Germination Conditions	Varieties	Value of Increase in Protein Digestibility In Vitro (%)
[29]	Soak at 22–25 °C for 12 h, germinate at room temperature under the wet cotton cloth for 24 h	Mung beans (*Phaseolus aureus*)	19.2%
		Cowpea (*Vigna catjang*)	14.3%
		Lentil (*Lens culinaris*)	14.5%
		Chickpea (*Cicer arietinum*)	14.3%
[5]	Germinated in the dark at ambient temperature for 72 h	Yellow pea (*CDC Amarillo*)	1.7%
		faba bean (*CDC Snowdrop*)	3%
[6]	Germinated in a seed germinator at 25 °C and 70% relative humidity for 20 h.	Mung bean	25%
		Chickpea	16.7%
		Cowpea	16.9%
[31]	Germinated at 28 ± 3 °C for 90 h	Chickpea (*NIFA-2005*)	55.9%
[35]	Germinated for 72 h at 30 °C at dark	Yellow field peas (*Pisum sativum L*., *CDC Centennial Cultivars*)	4%
[28]	placed on germination trays in a dark chamber set at 20 °C and 92% relative humidity for five days	Black beans (*Phaseolus vulgaris*)	13.3%

## Data Availability

No new data were created or analyzed in this study. Data sharing is not applicable to this article.
